# Oral contraceptive-related transverse sinus thrombosis as an initial manifestation of antiphospholipid syndrome in the absence of systemic lupus erythematosus

**Published:** 2017

**Authors:** Payam Saadat, Reza Mohseni-Ahangar

**Affiliations:** 1 Department of Neurology, Babol University of Medical Sciences, Babol, Iran.; 2 Babol University of Medical Sciences, Babol, Iran.

**Keywords:** Oral Hormonal Contraceptives, Lateral Sinus Thrombosis, Antiphospholipid syndrome, Anticardiolipin antibody, Antiphospholipid antibody.

## Abstract

**Background:**

Cerebral venous sinus thrombosis is a rare and potentially life-threatening neurologic manifestation of antiphospholipid syndrome. Oral contraceptive pills (OCP) may increase the risk of vascular events, even in people without family history of venous thrombosis.

**Case Presentation:**

A 31-year-old woman with four weeks of constant headache and history of taking OCP for one year has been selected for this study. The results of magnetic resonance imaging (MRI) of brain and venography confirmed a diagnosis of cerebral venous sinus thrombosis. The serum anticardiolipin and antiphospholipid antibodies were elevated and a definitive diagnosis of antiphospholipid syndrome was made.

**Conclusion:**

The present report demonstrates the importance of screening for antiphospholipid antibodies in patients presenting with cerebral venous sinus thrombosis despite history of taking OCP.

**C**erebral venous thrombosis (CVT) has been considered an uncommon disease with significant long-term morbidity and high mortality rate. It comprises approximately 0.5% to 1% of all strokes (1). CVT is a rare and potentially life-threatening neurologic manifestation of antiphospholipid syndrome (APS) (2).It has been estimated that 86% of cases involve the transverse sinuses (3). In the VTE literature, OC use in women is a well-recognized risk factor (4). Initially, estrogen in OC pills was thought to increase the clot risk. However, a study (5) has also linked progestins such as drospirenone and desogestrel, to VTE risk. The authors also demonstrate a dose-response relationship in the risk of CVT among women using OCs (6). In our country, the use of OCP has been increased especially in the last two decades and is available to married couples, free of charge at public clinics (7-8). Nonetheless, current conceptions displayed that many female patients took these drugs inappropriately without physician or health service approval. Also, many women use OCP for delaying menstruation to perform religious customs such as fasting in Ramadan (the month of fasting) and religious travels. In these cases, OCP are mostly being used in inappropriate time (menstruation cycle) or without physician consultation (9). We reported transverse sinus thrombosis as an initial manifestation of antiphospholipid syndrome in the absence of systemic lupus erythematosus in a 31-year-old woman who used OCP.

## Case Presentation

A 31-year-old woman was admitted to the emergency department with headache and vomiting. She had no history of aura and prodrome but reported blurred vision with no history of diplopia. The headache started about one month ago and was less frequent, but deteriorated and became constant at the day of admission. Headache first started in occipital region then radiated to all regions. Her headache aggravated by an activity and did not respond to common analgesics. She had a history of taking OCP for approximately one year.

**Figure 1:**
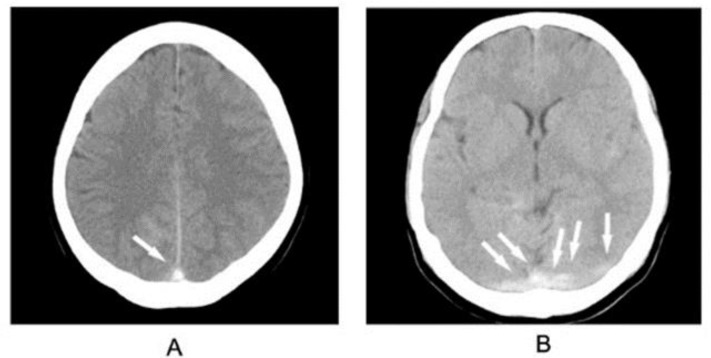
Brain non-enhanced computed tomography: dense triangle sign (A, arrow), dense clot sign (B, arrows

On admission, the patient appeared to be confused with slurred speech. Her vital signs were as follows: temperature 37.1°C, pulse rate 88/minute, blood pressure 90/70 mmHg, and respiration rate 21/minute. The neurological examination revealed bilateral, non-hemorrhagic papilledema especially in the left eye (stage 2). Visual fields and blind spots were normal. Likewise, the examination of the muscle strength of the limbs was 5/5 throughout and tone was within normal limits. The cerebellar testing could not be completed as the patient was uncooperative. In view of the condition, a brain computed tomography (CT) was performed and this revealed dense triangle sign and dense clot sign (figure 1). 

**Figure 2:**
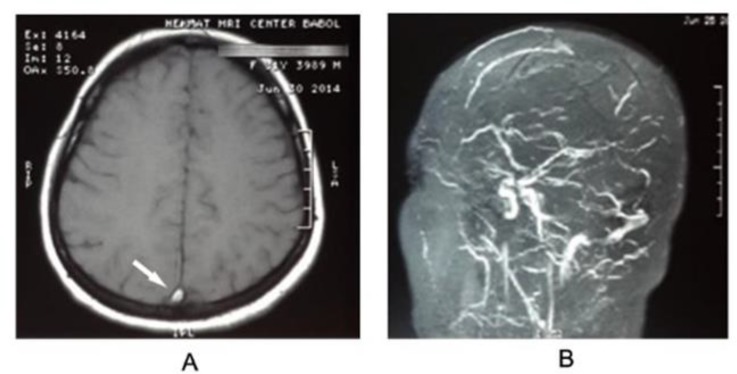
Magnetic resonance imaging of brain showed empty delta sign (A, arrowed) and magnetic resonance venography confirmed a diagnosis of cerebral venous sinus thrombosis (B

Magnetic resonance imaging (MRI) of brain was requested and empty delta sign was seen (figure 2A). Magnetic resonance venography (MRV) demonstrated extensive thrombosis in the transverse sinuses, and confirmed a diagnosis of cerebral venous sinus thrombosis (figure 2B).

The serum anticardiolipin antibodies were elevated: IgM 16 units/ml: (normal up to 7 units/ml). Besides, the antiphospholipid antibodies increased: IgM 21 units/ml (normal up to 10 units/ml). But rheumatoid factor (RF), anti-nuclear antigen (ANA), anti-double-stranded DNA (dsDNA), ANCA and antinuclear factor (ANF) were negative. From these results, a definitive diagnosis of antiphospholipid syndrome was made. 

## Discussion

Because of nonspecific and omnifarious clinical findings, the diagnosis of transverse sinus venous thrombosis can be difficult. Clinical manifestations of CVT depend on the location of the thrombosis. For transverse (lateral) sinus thrombosis, typical symptoms are headache and pain in the ear or mastoid region. Hemianopia, contralateral weakness, and aphasia may sometimes be seen due to cortical involvement (10). The only presentation of our patient was headache. Isolated headache without focal neurological findings or papilledema occurs in approximately 25 to 40 percent of patients with CVT, confusing the diagnostic process (11). 

We assume that the high titer of antiphospholipid antibodies, together with oral contraceptive use in our patient may raise the odds ratio for developing CVT. There has been suggested several causative conditions in etiology of cerebral venous thrombosis. These may be develop alone or in combination. Hypercoagulable states can be associated with the antiphospholipid syndrome that may result in CVT. APS is a prothrombotic condition including vascular obstructions in the presence of antiphospholipid antibodies. Antiphospholipid antibodies carry an increased risk for cerebral ischemic events and may therefore serve as a marker for imminent occurrence of thrombosis, especially in young individuals (12). It does appear that APS associated with CVT is rare. In addition, several medications specifically oral contraceptives (including the third-generation formulations) are related to an increased risk of CVT (13-15). Obviously, the great majority of young non-pregnant women with CVT are oral contraceptive users, and that the risk of CVT with oral contraceptive use in women is greater among those with a hereditary prothrombotic factor.

Two mechanisms have been suggested to the pathophysiology of cerebral venous thrombosis. First, when thromboses of cerebral veins or sinuses occur, cerebral perfusion decreased due to increased venular and capillary pressure. Decrease in cerebral perfusion leads to ischemic injury and cytotoxic edema. This could cause vasogenic edema, and capillary rupture finish with parenchymal hemorrhage. Second hypothesis proposed that cerebral sinus obstructions could cause decrease in the absorption of cerebral fluid, which resulted in increased intracranial pressure. Consequently, increased intracranial pressure worsens venular and capillary hypertension and contributes to parenchymal hemorrhage and vasogenic and cytotoxic edema (16). Very few cases of antiphospholipid syndrome have been reported from Iran. To the best of our knowledge, however, this is the first report of such a presentation in this region. This case demonstrates the importance of screening for antiphospholipid antibodies in patients presenting with cerebral venous sinus thrombosis. Primary antiphospholipid syndrome should be considered in the evaluation of patients with transverse sinus thrombosis, despite the presence of other risk factors like OCP use. 
